# Reproducibility of Corneal Graft Thickness measurements with COLGATE in patients who have undergone DSAEK (Descemet Stripping Automated Endothelial Keratoplasty)

**DOI:** 10.1186/1471-2342-12-25

**Published:** 2012-08-01

**Authors:** Melissa HY Wong, Annabel Chew, Hla M Htoon, Beng H Lee, Jun Cheng, Jiang Liu, Donald T Tan, Jodhbir S Mehta

**Affiliations:** 1 Singapore National Eye Centre (SNEC), 11 Third Hospital Avenue, 168751, SNEC, Singapore; 2 Singapore Eye Research Institute (SERI), SERI, Singapore; 3 Yong Loo Lin School of Medicine, National University of Singapore, Yong Loo Lin, Singapore; 4 Department of Clinical Sciences, Duke-NUS Graduate Medical School, Duke-NUS, Singapore; 5 Institute for Infocomm Research (I2R), Infocomm, Singapore

**Keywords:** Anterior segment optical coherence tomography, Descemet Stripping Automated Endothelial Keratoplasty, Graft thickness

## Abstract

**Background:**

The CorneaL GrAft Thickness Evaluation (COLGATE) system was recently developed to facilitate the evaluation of corneal graft thickness from OCT images. Graft thickness measurement can be a surrogate indicator for detecting graft failure or success. The purpose of this study was to determine the reproducibility of the COLGATE system in measuring DSAEK graft area between two observers.

**Methods:**

This was a prospective case series in which 50 anterior segment OCT images of patients who had undergone DSAEK in either eye were analysed. Two observers (MW, AC) independently obtained the image analysis for the graft area using both semi automated and automated method. One week later, each observer repeated the analysis for the same set of images. Bland-Altman analysis was performed to analyze inter and intra observer agreement.

**Results:**

There was strong intraobserver correlation between the 2 semi automated readings obtained by both observers. (r = 0.936 and r = 0.962). Intraobserver ICC for observer 1 was 0.936 (95% CI 0.890 to 0.963) and 0.967 (95% CI 0.942 to 0.981) for observer 2. Likewise, there was also strong interobserver correlation (r = 0.913 and r = 0.969). The interobserver ICC for the first measurements was 0.911 (95% CI 0.849 to 0.949) and 0.968 (95% CI 0.945 to 0.982) for the second. There was statistical difference between the automatic and the semi automated readings for both observers (p = 0.006, p = 0.003). The automatic readings gave consistently higher values than the semi automated readings especially in thin grafts.

**Conclusion:**

The analysis from the COLGATE programme can be reproducible between different observers. Care must be taken when interpreting the automated analysis as they tend to over estimate measurements.

## Background

Descemet stripping automated endothelial keratoplasty (DSAEK) is rapidly becoming an alternative to penetrating keratoplasty (PK) for patients with corneal endothelial failure [1]. The procedure involves the formation of a 50–150 micron button of donor posterior lamellar tissue which is used to replace the diseased corneal recipient endothelium and Descemet membrane. DSAEK is considered to have better tectonic safety and more favourable post-operative refractive outcomes compared to PK [1].

Optical Coherence Tomography (OCT) is an infra red light imaging device that captures micrometer resolution, three dimensional images of the anterior segment [2]. Examples of current systems that use spatial domain technology are the Stratus OCT (Carl Zeiss Meditec Inc, Dublin, CA), Visante anterior segment OCT system (Carl Zeiss Meditec Inc, Dublin, CA) and slit lamp OCT (Heidelberg Engineering, Heidelberg, Germany) which are time domain OCTs. As a result of involuntary eye movements, the images acquisition are usually of reduced resolution compared to newer systems using fourier domain technology [3,4]. These include the Cirrus HD OCT (Carl Zeiss Meditec, Inc.), Spectralis® HRA + OCT (Heidelberg Engineering, Inc) and RTVue (Optovue, Inc). However, the disadvantage of the higher resolution machines is that they do not allow complete image acquisition of across the entire anterior chamber [3]. The image resolution of the various OCT machines are compared in Table 1.

**Table 1:** Differences between the various Optical Coherence Tomography (OCT) machines

	**Stratus OCT**	**Visante OCT**	**SL- OCT**	**RTVue FD OCT**	**Cirrius HD OCT**
Manufacturer	Carl Zeiss Meditec	Carl Zeiss Meditec	Heidelberg	Optovue	Carl Zeiss Meditec
Axial Resolution	10um	18um	<25um	5um	5um
Scan Speed	400 A scans per sec	2000 A scans per sec	200 A scans per sec	26000 A scans per sec	27000 A scans per sec

**Figure 1:**
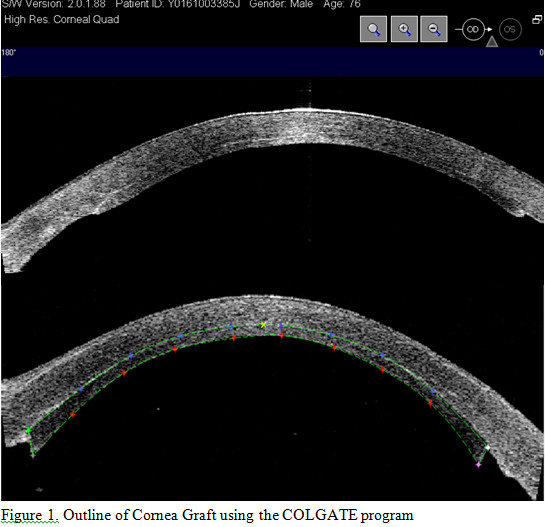
Outline of Comea Graft using the COLGATE program.

Users are able to quantify the images captured with the OCT using the built-in caliper software. For corneal graft thickness measurements the user can manually adjust the calipers to obtain thickness measurements at different locations along the graft. With individual point caliper measurements, the user only obtains information at a selective point on the graft and information about the complete graft thickness profile is not obtained. In order to circumvent this problem, a semi-automated system, the COrneaL GrAft Thickness Evaluation (COLGATE) software program was developed to facilitate the evaluation of corneal graft on OCT images. The COLGATE system [5] automatically detects the boundaries of the graft according to the best fit curve of the program. The observer then has the option to manually adjust the margins of the curve to enhance the alignment with the graft profile (Figure 1). The software subsequently calculates the area of the graft within the demarcated points in square microns thereby providing the complete graft thickness profile from the OCT image. Measuring the complete graft area is a more accurate way of documenting graft thickness following transplantation as graft thickness can be variable following automated lamellar therapeutic keratoplasty [5,6].

The aim of this study was to evaluate the inter-observer and intra-observer reproducibility of DSAEK graft area measurements using both automated and semi-automated methods of the COLGATE program. The results from this study allows for validation of the software for future studies.

## Methods

### Subjects

In this retrospective study, 50 high resolution cornea OCT images of 23 patients who had undergone DSAEK in either eye were obtained from a common data base. The data base encompasses all anterior segment OCT images which are kept in the hard disk drive of the OCT machine These 50 images were selected as they had the best quality images with both cross-sectional ends of the DSEK graft seen. There were 10 females and 13 males ranging in age from 42 to 83 years old, with mean age of 62.7 years old +/− 17.6. Of these, 52.2% were Chinese, 17.4% were Malays, 17.4% were Vietnamese, 8.7% were Indians and 4.3% were Eurasians. The study had the approval of the hospital’s Ethics Committee (Singhealth Institutional Review Board) and was conducted according to the tenets of the Declaration of Helsinki.

### Image acquisition

The AS- OCT (anterior segment optical coherence tomography) (Visante, Carl Zeiss Meditec, Dublin, California) images of the anterior chamber were obtained using high resolution cornea images in a completely dark room with no windows and the only lighted areas were the fixation target which equates to 20 lux illumination. To obtain the best quality image, the examiner adjusted the saturation and noise and optimized the polarization for each scan during the examination so as to obtain good discernible images with high signal to noise ratio. The patients were instructed to fixate at the external fixation light to ensure that they were looking straight ahead. The patients’ eye lids were kept open so that they did not block the 10-mm diameter corneal mapping. The operator adjusted the software system to position the vertex at the center of the AS-OCT image and to maximize the vertex reflection. The images were obtained by the same operator for all 50 eyes. All scans were taken between 10 AM and 4 PM to minimize the effect of diurnal variation on cornea thickness. More than one horizontal scan was performed but the best quality scan was selected by the technician performing the scan, for measurements.

### Image processing and analysis

**Figure 2:**
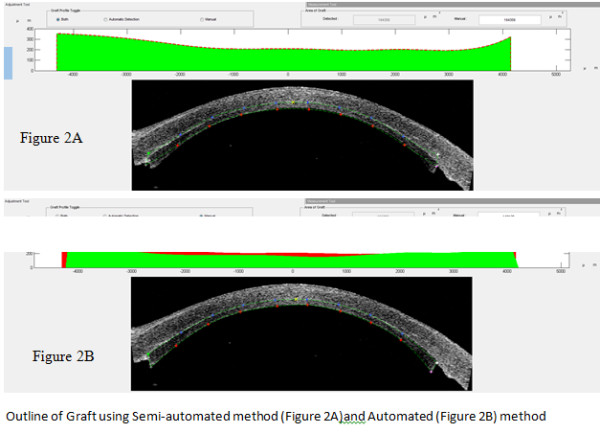
**Outline of graft using semi-automated method (Figure **2**A) and automated (Figure **2**B) method.**

The images were processed through the COLGATE program that analyses the images via four main steps as previously described [5]. The first step involves extraction of the boundary of the corneal graft OCT image. The images are first filtered and converted to a binary image through the Canny edge detector [7] which uses an algorithm to detect a range of edges in an image. The output is then sampled in steps of 10 pixels so that the effects of the interference noise on the extracted boundary are reduced. The resulting refined points allow for a smoother and more accurate boundary for graft segmentation. The second step locates 4 corner points on the transplanted graft based on global and local curvature properties [8]. The third step extracts points that lie on the boundary between the patient’s original cornea and the graft to create a best fit curve. (Figure 2A) However as there is little contrast of this boundary with the surroundings, accurate detection of the boundary can be difficult. The points on the anterior border, together with the corner points detected in the earlier step and the information from the posterior border of the corneal graft in the boundary detection mode, provides enough information to segment the graft. A profile of the graft thickness is displayed to allow the user to better evaluate the graft. After the automated graft detection process, the user can further refine the detected graft by manually adjusting the control points on the graft [5]. (Figure 2B).

### Repeatability of image analysis

The definition of reproducibility used in this study was based on definitions by the International Organization for Standardization [8,9]. Two ophthalmologists (MW, AC) independently performed the image analysis using the COLGATE program on the same set of 50 high resolution cornea images selected and downloaded from the Visante OCT machine. The two individuals were taught how to use the software but it was the first time using it on the 50 ASOCT images. They used the COLGATE program to obtain the best fit curve over the entire graft. (Figure 2A) The graft area under the automated curve was recorded. Each ophthalmologist then manually readjusted the 4 corner points as well as points on the anterior and posterior margins of the graft to create a best fit curve for the graft. This second reading was recorded down. One week later, each observer repeated the analysis for the same set of images independently. They were masked to the results of the initial analysis taken a week earlier, as well as to the measurements of the other observer. These measurements were then recorded and compared against the automated set of readings.

### Statistical methods

Bland-Altman analysis was performed to analyze inter and intra observer as well as between automated and semi-automated agreement using MedCal Version 12.3.0 (Mariakerke, Belgium). The reproducibility of the above parameters was calculated in terms of limits of agreement (LOA; mean of differences +/− 1.96x standard deviation of differences). Paired t-tests were used for the differences between observer measurements.

## Results

Both observers were able to define the outline of the graft for all 50 images and calculate the graft area in an automated and semi automated method, on two separate occasions.

### Intraobserver repeatability

The first and second sets of readings were similar within each observer as evident by strong linear correlation coefficient values (r = 0.936 and r = 0.962) as well as intraobserver correlation coefficient values (ICC). The intraobserver inter class correlation coefficient (ICC) for observer 1 was 0.936 (95% CI 0.890 to 0.963) and observer 2 was 0.967 (95% CI 0.942 to 0.981). This implies that overall the two sets of readings made by each observer not only strongly resemble each other (coefficient value) but each observer was able to obtain a point to point similarity between the two sets for readings of the calculated graft area for all 50 images (ICC). The mean difference of each of the calculated graft areas by the semi automated method, was expressed as the mean of the limits of agreement (LoA mean) and this was smaller for observer 1 ie −223.8 um2 (−4340.6 to 3893.1) than for observer 2 at −3477.5 um2 (−6695.5 to −259.5). (Table 2).

**Table 2:** Difference in intraobserver mean area calculation

				**Bias (95% CI)**		
	**Mean area (um2) ±SD**	**P value**	**R value ICC value**	**LoA Mean**	**Upper limit**	**Lower limit**
M1	151619.4 ± 40912.0	0.914	0.936 0.936 (95% CI 0.89-0.963)	−223.8 (-4340.6 to 3893.1)	28168.4 (21085.6 to 35251.3)	−28616.0 (-35698.8 to -21533.1)
M2	151843.2 ± 39172.1					
B1	148255.0 ± 39651.0	0.035	0.962 0.958 (95% CI 0.925-0.977)	−3477.5 (-6695.5 to -259.5)	18715.9 (13179.4 to 24252.4)	−25671.0 (-31207.5 to -20134.5)
B2	151732.6 ± 41381.0					

### Interobserver repeatability

The semi automated graft area calculation readings were compared between the two observers and this was found to be comparable between the two groups. This relationship (between calculations made by observer 1 and observer 2 for the two sets) was reflected in the linear correlation values (r = 0.913 for the first set and r = 0.969 for the second set) and there was no statistical significance in the calculations made between the two observers for each set of readings. This was further supported by the interobserver interclass correlation coefficient value (ICC) for the first set of measurements between observer 1 and observer 2 (0.911 (95% CI 0.849 to 0.949)) and the second set of measurements 0.968 (95% CI 0.945 to 0.982). This means that there was point to point agreement of the calculated graft area obtained between observer 1 and observer 2 for all 50 graft area calculations for both sets of readings. The difference of each calculated graft area (for 95% of all the observations) by observer 1 and observer 2 from the mean of both their calculations is again expressed as the mean LoA. The mean LoA for the first set of readings (between M1 and B1) was 3364.4um2 and even was even smaller for the second reading (between M2 and B2) at 110.6 um2 (Table 3).

**Table 3:** Difference in interobserver mean area calculation

				**Bias (95% CI)**		
	**Mean area (um2) ±SD**	**P value**	**R value ICC value**	**LoA Mean**	**Upper limit**	**Lower limit**
M1	151619.4 ± 40912.0	0.164	0.913 0.911 (95% CI 0.849-0.949)	3364.4 (-1423.0 to 8151.7)	36381.0 (28144.5 to 44617.4)	−29652.2 (-37888.7 to -21415.7)
B1	148255.0 ± 39651.0					
M2	151843.2 ± 39172.1	0.940	0.969 0.968 (95% CI 0.945-0.982)	110.6 (-2802.0 to 3023.2)	20197.8 (15186.7 to 25208.8)	−19976.5 (-24987.6 to-14965.5)
B2	151732.6 ± 41381.0					

### Comparison between automated and semi-automated

Four sets of readings between the automated and the semi automated method (M1, M2, B1, B2) were compared. In the first set (automated versus M1), there was statistical difference in the mean graft area calculated by the automated method (195474.6um2) and semi-automated 151619.4um2 by observer 1(p = 0.006). The area calculated by the automated method and the first set of calculations made by the first observer (M1) were not correlated r = −0.083. The LoA mean in this first group was 43855.14 um2 implying that 95% of all calculated graft areas of the 50 images using the automated and semi automated method were found to be far from the mean of the calculated automated and semi automated graft area.

In the second set, the graft area calculated by the automated method was 195474.6um2 and semi-automated 151843.2um2 (M2) (p = 0.006). Again, the two sets of results were not correlated (r = −0.08) and the large LoA mean of 43631.38 um2.

Observer 2 also had 2 sets of graft area calculations compared with the automated method. For the first set of readings done by semi automated method (B1), observer 2 obtained 148255.0 um2 for the mean graft area while the automated method had a reading of 195474.6um2 (p = 0.003). The calculated area of each image obtained by the automated method and observer 2 were not correlated (r = −0.08). Likewise, the large LoA mean of 47219.5 um2 implies that 95% of all calculated graft areas by automated and semi automated method (B1) were far from the mean of the automated and semi automated graft area values (B1). For the second set of calculations made by observer 2 (B2), observer 2 obtained 151732.6um2 and this was statistically significantly different (p = 0.006) from the automated value. Once again, there was a negative linear correlation of r = −0.05 and a correspondingly large limits of agreement between automated and B2 values (LoA mean 43742 um2). Table 4 shows the comparison between automated and the mean of each of the observers’ calculations. In both observers, the automated method gave consistently larger values than the semi automated method.

**Table 4:** Difference between automated and semi-automated mean area calculations

				**Bias (95% CI)**		
	**Mean area (um2) ±SD**	**P value**	**R value**	**LoA Mean**	**Upper limit**	**Lower limit**
Automated	195474.6 ± 96311.3	0.006	−0.062	43187.1 (13056.0 to 73318.3)	250990.3 (199150.8 to 302830.0)	−164616.1 (-216455.7 to -112776.5)
S.A M(mean of M1, M2)	152287.4 ± 38750.8					
Automated	195474.6 ± 96311.3	0.004	−0.058	45885.2 (15590.3 to 76180.1)	254817.6 (202696.4 to 306938.9)	−163047.2 (-215168.5 to -110926.0)
S.A B(mean of B1 ,B2)	149589.4 ± 40447.4					

## Discussion

There is much subjective variation in the software caliper placement by users of the ASOCT, and inter-observer variations of measurements have been shown to have a SD of 18.0 - 20.2um at +1.0 mm and −1.0 mm from the centre of the cornea.* Recently we compared the interobserver and intraobserver variation of LASIK flaps measured using a time domain and spectral domain machine [6]. The interobserver and intraobserver results for the time domain machine were similar to that previously published [2]. The mean limit of agreement (LOA) was worse for the central cornea reading compared to those measurements taken at +1.5 mm and −1.5 from centre. Using the time domain OCT, the interobserver correlation coefficients (r) were 0.73(−1.5 mm from centre), 0.62 (centre) and 0.78 (+1.5 mm from the centre). For spectral domain machines the LOA was much closer for the two observers, and interobserver correlation coefficients were much stronger 0.82 (−1.5 mm from the centre), 0.88 (centre) and 0.88 (+1.5 mm from the centre) [6]. Hence the improved resolution allowed for improved accuracy in measurements, both inter and intra-observer. However, these are only single point measurements and to get multiple point information an automated/semi-automated system would be more efficient, faster and reduce the inherent error rate.

In this study, we found that the COLGATE program was a highly reproducible tool for graft area measurements and consistent graft area calculations could be obtained both inter and intra-observer from every scan. This was evident by the strong linear correlation coefficient, intraclass and interclass coefficient and the small range of limits of agreement. It was observed that the automated method gave consistently higher values than the semi automated method and this was especially pronounced in thin grafts. In particular graft areas of less than 80000um^2^ measured by the semi automated method had more than double the measurement by the manual method. This may be due to the fact that the boundary between the anterior graft surface and the underside of the recipient stroma might not be as easily discernible in thin grafts by the COLGATE program. The authors believe that using the semi automated method to calculate the graft area would be better in all grafts.

We found a higher linear correlation of the graft area measurement made between observers (r = 0.913, r = 0.969) using the COLGATE program than the previously reported inter-observer correlation on the same time domain ASOCT (r = 0.841, r = 0.751) [6]. The automated/semi-automated system has several advantages over multiple single point measurements as the entire graft thickness is taken into consideration. An alternative would be to take an intergral of multiple points on the graft but the latter option would be laborious and it is not physically possible to include every single point from the graft border. Using the automated system initially allows rapid delineation of the graft boundaries that are identified by the program and this gives an approximate estimate of the graft border. The user is then able to fine-tune the measurements by adjusting the software to delineate the graft border more accurately. This semi-automated programme is akin to the Heidelberg Retina Tomograph software^#^ used in glaucoma for evaluating the optic nerve head or the Artemis high frequency ultrasound system [10].

The COLGATE system may be used as an objective method for ophthalmologists and researchers to obtain graft area measurements. This raises the possibility that DSAEK surgeons may then have a program to preoperatively predict the maximum donor diameter of the graft to be inserted so as to enable transfer of the maximal amount of donor endothelial cells, and with a chart including a range of individual donor thicknesses, be able to select and decide on the exact diameter required at the time of surgery taking into account the actual thickness of the donor tissue supplied for the case, without the fear of encroaching into the chamber angle and risking donor iris contact at the periphery.

Currently, graft thickness is measured using the software calipers on the Visante ASOCT (as well as other OCT systems) at a single point. However, as most grafts are irregularly cut, the central thickness does not provide a good estimate of the entire graft thickness [11]. There is often a mismatch in thickness between the central and peripheral graft of between 75 to 100 microns [12]. The Optovue system also utilizes similar caliber software in a similar manner with similar potential errors of measurement. The post op DSAEK total corneal thickness is a surrogate marker for the physiological ‘well-being’ of the donor corneal allograft as a thin graft implies a healthy endothelium and clear cornea free of corneal edema. In corneal grafts with good endothelial function, the grafts are often thinner compared to thickness measurements in the early postoperative period. In cases of early or late graft failure or in cases of graft rejection the graft will become swollen, and thicker. It is believed that as early as one week post DSAEK the surgeon is able to predict the likelihood of graft survival based on ASOCT measurements of the central and peripheral cornea thickness. [13]. Authors have found that failed DSAEK grafts were significantly thicker at post operative week one onwards compared to successful grafts. There has also been increasing interest in the relationship between DSAEK graft thickness and post-operative refractive error. There have also been other reports on how the difference in thickness between center and periphery of the DSAEK graft induces a change in posterior corneal curvature resulting in a hyperopic shift [14-16]. Hence knowing the post operative graft thickness will allow the surgeon to better visually rehabilitate the patient with future graft refractive procedures.

There are other uses of this software. Its use can be extended to evaluating penetrating keratoplasty grafts, as well as anterior and deep anterior lamellar grafts. Future software enhancements will also allow it to be possible to measure the anterior and posterior curvature of the graft, which are useful parameters in assessing post graft refractive evaluation [15]. Currently this is not possible with any other software and can only be determined from supportive data [14-16]. The inbuilt automatic boundary detector can also be extrapolated to evaluate iris profile as well as measurements of the trabecular iris surface area (TISA) [17] in glaucoma patients.

There are some limitations to this program. Though the graft area is a better surrogate for graft thickness than single point measurements as it incorporates the entire graft, the graft volume would be a more precise parameter than area. However, this would require a three-dimensional imaging acquisition of the corneal graft, which currently is not available. An estimation of graft volume may be calculated from our values by multiplying the graft area by the graft diameter which will be known to the surgeon implanting the graft e.g. if area is 20000 um^2^, volume = area x size of trephine (ie 8.5 mm) = 170,000um^3^ It is also not sufficient to simply rely on the automated readings as we found that the semi automated readings gave a better and consistent graft area calculation.

Lastly, it would also be interesting to demonstrate how image quality changes the inter-observer and intra-observer reproducibility of the measurements.

## Conclusion

This paper shows that the intra-observer and inter-observer repeatability from the COLGATE program is highly reproducible, and could be used clinically to determine the ideal diameter of a donor lenticule for DSAEK surgery. However care must be taken when interpreting the automated analysis as it tends to over estimate corneal graft area, especially in thinner grafts. Nevertheless, the semi-automated program is useful in evaluating the graft area which in turn is a better indication of the graft thickness and hence graft function. Further studies evaluating graft thickness and graft refractive power longitudinally postoperatively using this software are planned. Knowing the expected hyperopia from the calculated graft thickness can help the surgeon determine the refractive end point especially when performing a combined DSEK with phacoemulsification.

## Endnotes

^a^Visante Operation Manual. Jena, Germany, Carl Zeiss Meditec AG, 2006;appendix D-6:158.

^b^http://www.heidelbergengineering.com.

## Competing interests

The authors report no conflicts of interest.

## Authors’ contributions

JM, DT: Initiating the study and tutoring MW and AC. MW and AC contributed to the acquisition of images using the software. MW: Writing of the manuscript. H Htoon assisted with statistical analysis. JLee, J Cheng and B Liu: contributed to the analysis and creation of the COLGATE software. All authors have read and approved the final manuscript.

## Pre-publication history

The pre-publication history for this paper can be accessed here:

http://www.biomedcentral.com/1471-2342/12/25/prepub
